# Methylprednisolone Pulse Therapy or Additional IVIG for Patients with IVIG-Resistant Kawasaki Disease

**DOI:** 10.1155/2020/4175821

**Published:** 2020-11-24

**Authors:** Zhouping Wang, Feiyan Chen, Yanfei Wang, Wei Li, Xiaofei Xie, Peiying Liu, Xu Zhang, Li Zhang, Ping Huang

**Affiliations:** ^1^Department of Cardiology, Guangzhou Women and Children's Medical Center, Guangzhou Medical University, Guangzhou, Guangdong, China; ^2^Department of Pediatric Intensive Care Unit, Guangzhou Women and Children's Medical Center, Guangzhou Medical University, Guangzhou, Guangdong, China

## Abstract

There have been no robust data from clinical trials to guide the clinician in the choice of therapeutic agents for the child with intravenous immunoglobulin (IVIG) resistance. The treatment regimen for IVIG-resistant patients varies between institutions, and the best option has not yet been established. Therefore, in this trial, a total of 955 patients with Kawasaki disease (KD) were selected and were initially treated with IVIG (2 g/kg), of whom 80 (8.38%) assessed as IVIG resistant were randomly divided into two groups: Group A received the second IVIG treatment (*n* = 40), and Group B received methylprednisolone pulse therapy (MPT, *n* = 40). The whole fever time, duration of fever after retreatment, hospital days, medical costs, readmission rate, and laboratory examination difference (△) were calculated. Coronary artery lesion (CAL) outcomes were followed up over two years. Patients in the MPT group had shorter fever after retreatment and lower medical costs; more rapid declines in C-reactive protein (CRP), neutrophils (N%), and platelet (PLT) levels; and more rapid rise in sodium. However, they also probably had a higher incidence of treatment failure and CALs than the additional IVIG treatment group in the long-term follow-up. Caution is still required in the use of MPT to treat IVIG-resistant KD.

## 1. Introduction

KD is an acute, self-limited febrile illness of unknown cause that predominantly affects children < 5 years of age [1]. It has been reported worldwide and is the leading cause of acquired heart disease in children in developed countries [2]. The efficacy of IVIG administered in the acute phase of KD is well established to reduce the prevalence of CALs [3]. However, approximately 10% to 20% of patients with KD who do not respond to the initial treatment with IVIG at least 36 h after the end of their IVIG infusion are termed IVIG resistant [4]. It is well known that those with IVIG-resistant KD have a higher risk for the development of CALs [5, 6]. The treatment regimen for IVIG-resistant patients varies between institutions, and the best option has not yet been established [7]. IVIG-resistant patients are usually treated with additional IVIG. Retrospective series have suggested efficacy, but IVIG retreatment has never been tested in an adequately powered randomized trial [8, 9]. Consequently, IVIG retreatment for IVIG-resistant KD is far from an established therapy. Corticosteroids have also been used to treat patients who have failed to respond to initial IVIG therapy for KD [10]. Several retrospective studies suggest that MPT for IVIG-resistant KD may reduce the risk for CALs [11, 12]. There have been no prospective randomized controlled clinical trials (RCTs) comparing MPT with a second dose of IVIG for IVIG-resistant KD patients, and there are no robust data from clinical trials to guide the clinician in the choice of therapeutic agents for the child with IVIG resistance [13]. Therefore, we conducted a prospective RCT to determine whether patients with initial IVIG-resistant KD may benefit from MPT than from additional IVIG.

## 2. Methods

### 2.1. Trial Design

This was a prospective, single-center, RCT. A total of 955 patients with KD at the Department of Cardiology of Guangzhou Women and Children's Medical Center from January 2018 to June 2019 were selected and initially treated with IVIG. 80 patients who were assessed as IVIG resistant were randomly divided into two groups using a random number table. The Research Ethics Committee of Guangzhou Women and Children's Medical Center approved this study (No. GZR2015-099), and the children and their parents provided their written informed consent. The registration number of this randomized controlled clinical trial is ChiCTR-EOC-17013266.

### 2.2. Sample Size

The sample size was 80 cases with 40 cases in each group. Use was made of the formula [14] *n* = (*μα* + *μβ*) 2*σd*2/*δd*2, where *μα* and *μβ* can be found in the *μ* value table [14], *σd* is the standard deviation of the difference, and *δd* is the difference. After consulting the table, *μα* = 1.960, *μβ* = 1.280, *σd* = 0.038, and *δd* = 0.028. The research group conducted a pretest, and 45 samples were calculated for each group. The overall calculation was obtained with the assumption of a 10% dropout rate.

### 2.3. Grouping Method

A random number table method was used to allocate the participants to a group. Nonparticipating nurses used a computer to generate random tables. Once eligible subjects had entered the trial, the nurses assigned the groups to trial researchers for clinical trial intervention.

### 2.4. Blind Method

The trial did not employ any blinding.

### 2.5. Patient Involvement Statement

We recruited IVIG-resistant KD patients from the Department of Cardiology of Guangzhou Women and Children's Medical Center between January 2018 and June 2019. For patients who met the inclusion criteria, a research project description was provided to their family, and written consent was obtained. The parents were involved in the research and actively contributed to identifying the issue of inconsistent reporting, the need for guidance, and the research question. The parents were involved as research partners in all aspects of the study, including determining the design of this study, determining the original research question, determining the need for therapy, and identifying the need for consensus. The research reports and results will be disseminated to all study participants via email.

### 2.6. Diagnosis Criteria of KD

The diagnosis of KD was made according to the guidelines of the diagnosis, treatment, and long-term management of Kawasaki disease established by the American Heart Association (AHA) in 2017 [13].

### 2.7. Diagnosis Criteria of IVIG-Resistant KD

KD patients who developed recrudescent or persistent fever at least 36 h after the end of their first IVIG infusion were identified as IVIG resistant [13].

### 2.8. Diagnosis Criteria of CALs

We use the maximal *Z* scores for the evaluation of the severity of CALs. The *Z* scores from echocardiographic assessment of luminal dimensions are classified into five categories [13] as follows:

1. No involvement: *Z* scores always <2.
2. Dilation only: *Z* score ≥ 2 but <2.5.
3. Small aneurysm: *Z* score ≥ 2.5 to <5.
4. Medium aneurysm: *Z* score ≥ 5 to <10, and absolute dimension < 8 mm.
5. Giant aneurysm: *Z* score ≥ 10, or absolute dimension ≥ 8 mm.

### 2.9. Inclusion Criteria of KD Patients

The inclusion criteria include KD patients diagnosed and hospitalized in our hospital who met the diagnostic criteria for KD.

### 2.10. Exclusion Criteria of KD Patients

The following are the exclusion criteria of KD patients:

1. The presence of other diseases that affect the temperature change in the course of the KD, such as sepsis, influenza, and juvenile idiopathic arthritis
2. The absence of detailed information about their initial treatment outside the hospital
3. A prior history of KD
4. Treatment with hormone or immunosuppressant therapy in the preceding 30 d
5. The presence of a severe immune disease, such as immunodeficiency or chromosomal abnormality
6. Refusal to sign informed consent
7. Inability to follow up for at least 6 mo

### 2.11. Trial Withdrawal and Termination Criteria

The following are the trial withdrawal and termination criteria:

1. If participants voluntarily withdraw informed consent at any time during the study, they were withdrawn from the trial and excluded from data analysis
2. Were either of the following conditions to occur, the patient's testing was to be terminated: the patient gives up treatment; fever is more than 36 h after retreatment

### 2.12. The Definition of Readmission

Patients admitted to the hospital within 3 d of discharge were treated as a readmission.

### 2.13. Initial Treatment

All patients meeting the diagnostic criteria for KD were initially treated with high-dose IVIG (2 g/kg given as a single intravenous infusion) together with aspirin orally (30-50 mg/kg/d) within the first 10 d of the onset of fever. We defined a responder as a patient who showed resolution of fever (<38°C) within 36 h after initial IVIG treatment [15].

### 2.14. Additional IVIG Treatment and MPT

IVIG-resistant patients were randomly divided into two groups by a random number table: Group A received additional IVIG (2 g/kg given as a single intravenous infusion), and Group B received MPT (methylprednisolone 15 mg/kg/d intravenously for 3 d, without a subsequent course and taper of oral prednisone). Both groups took aspirin orally (30-50 mg/kg/d) [12]. During MPT and IVIG treatment, patients underwent continuous cardiac monitoring. Frequent evaluations were made after the infusion until vital signs were stable.

### 2.15. Observation Indicators

The laboratory values of white blood cells (WBC), N%, CRP, hemoglobin (HGB), PLT, albumin, and sodium 1 d before and 1 wk after retreatment were measured, and the difference before and after treatment was calculated (△). The duration fever time (total days from the onset of fever to stable temperature for 48 h after treatment), duration of fever after retreatment (time required to stabilize body temperature for 48 h from the start of retreatment), hospital days (a period from a patient's first admission date to a discharge date after retreatment), medical cost from admission to discharge, readmission rate, and CALs for the two groups were compared.

### 2.16. CALs

The incidence of CALs was assessed at 7 days after diagnosis and followed up at 1, 3, 6, 12, and 24 mo after hospital discharge using *Z* scores from ultrasound echocardiography according to the diagnosis criteria of CALs [13]. The minimum follow-up time is approximately 12 mo.

## 3. Data Management

Relevant quality control was carried out throughout the trial process to ensure the authenticity, accuracy, and objectivity of the paper data. Two people entered the electronic data and locked the data for storage.

## 4. Statistical Analysis

Data analysis was carried out using SPSS19.0 software. Measurement data consistent with a normal distribution are expressed as *x* ± *s*, and comparison between groups was made using *t*-tests. For the statistical analysis, the Kruskal-Wallis test was used for paired numeric data, the Mann–Whitney *U* test was used for unpaired numeric data, and the chi-squared test or fisher's exact test was used for categorical variables. A *P* value lower than 0.05 by two-tailed analysis was accepted as statistically significant.

## 5. Treatment Results

**Figure 1:**
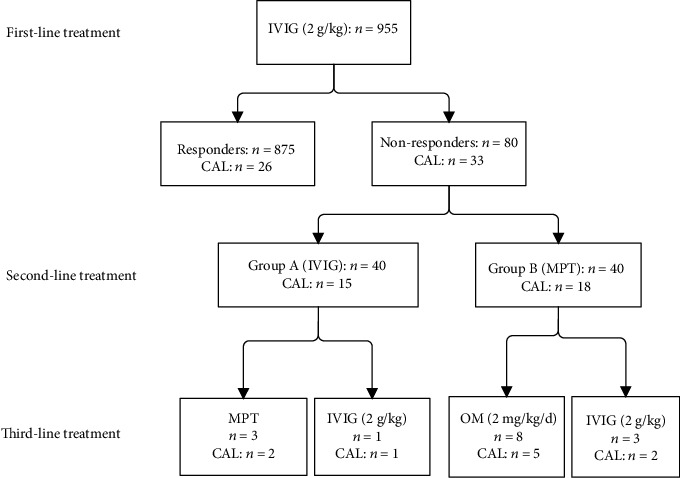
Protocol and results of the treatment in this study. IVIG: intravenous immunoglobulin; CAL: coronary arterial lesion; MPT: methylprednisolone pulse therapy; OM: oral methylprednisolone.

As shown in Figure 1, 955 patients with KD were treated with IVIG (2 g/kg) immediately after being diagnosed with KD. Of these, 875 (91.62%) were clinical responders to the initial treatment, and 26 (2.97%) showed CALs. However, the remaining 80 patients (8.38%) were assessed as IVIG resistant, and 33 of these (41.25%) showed CALs. In Group B, 35 patients (87.5%) responded to MPT and 5 (12.5%) did not. In Group A, fever was alleviated by additional IVIG in 34 patients (85%) but not in the remaining 6 (15%). We administered third-line therapy to those who did not respond to the second-line treatment: eight of the eleven patients who were readmitted to hospital received oral methylprednisolone (2 mg/kg/d), and three received IVIG (2 g/kg). Three of the four patients who were resistant to additional IVIG received MPT (methylprednisolone 15 mg/kg/d intravenously for 3 d), and one was treated again with IVIG (2 g/kg). There was no significant difference in the CAL rate between the two groups (75%, 3/4 vs. 63.6%, 7/11; *P* < 0.05). Five patients developed bradycardia in the MPT group, but their conditions improved spontaneously. In the additional IVIG treatment group, there were no adverse reactions occurred.

As shown in Table 1, no significant difference was found between the two groups with regard to age, sex, weight, hospital days, or duration of fever. The duration of fever (≥38°C) after additional treatment in patients treated with MPT (11 ± 6.3 h) was significantly shorter than that in patients treated with additional IVIG treatment (18 ± 4.4 hours; *P* < 0.05). A significant difference was found in medical cost between the two groups (¥11261 ± 3564 in Group A versus ¥8218 ± 2145 in Group B; *P* < 0.05), but the readmission rate in patients treated with MPT (27.5%, 11/40) was significantly higher than that in patients treated with additional IVIG treatment (10.0%, 4/40; *P* < 0.05).

The differences in laboratory data between 1 d before and 7 d after additional treatment are shown in Table 2. No significant difference was found between the two groups in △WBC, △HGB, and △albumin, but △N%, △CRP, △PLT, and △sodium after additional treatment in patients treated with MPT were significantly greater than those in patients treated with additional IVIG treatment (*P* = 0.048, *P* = 0.039, *P* = 0.027, *P* = 0.043, and *P* < 0.05, respectively).

As shown in Table 3, there was no statistically significant difference in the incidence of CALs between the two groups at 7 days (*P* = 0.650, *P* > 0.05), and no statistically significant difference at 1 mo, 3 mo, and 6 mo after hospital discharge (*P* = 0.202, *P* > 0.05; *P* = 1.000, *P* > 0.05; and *P* = 0.674, *P* > 0.05, respectively). However, at 12 mo and 24 mo follow-up, the MPT group probably had a higher incidence of CALs than did the IVIG treatment group (*P* = 0.043, *P* < 0.05; *P* = 0.033, *P* < 0.05).

Because the patients with KD in our hospital come from all over the country, as time goes by, those patients who did not have CALs in the previous examinations will not return to the hospital for examination, so more and more patients are dropout. But there were no CAL-positive KD patients among the dropouts. At 6 months after discharge, one patient in Group B who had no CALs previously had a small aneurysm, so the number of Group B CAL-positive cases increasing from that in 3 months to 6 months.

## 6. Discussion

In the current RCT with 955 KD patients and 80 IVIG-resistant KD patients, we verified that the medical costs of patients treated with MPT were significantly lower and the duration of fever after retreatment was significantly shorter than those of patients treated with additional IVIG treatment. In addition, the WBC, PLT, Na, and N% of patients treated with MPT returned to normal faster than those of patients treated with additional IVIG treatment. However, MPT probably had a higher incidence of treatment failure and CALs than the IVIG retreatment group in long-term follow-up. To the best of our knowledge, this is the first prospective RCT to study MPT and additional IVIG for patients with IVIG-resistant KD in China. We define patients who need to be readmitted to the hospital within 3 d of discharge as treatment failure. The treatment failure rate in patients treated with MPT (27.5%, 11/40) was significantly higher than that in patients treated with additional IVIG treatment (10.0%, 4/40; *P* < 0.05). Those patients were classified as no-responders to retreatment, and we administered third-line therapy to those patients. Eight of the eleven readmitted patients received oral methylprednisolone (2 mg/kg/d), and three received IVIG (2 g/kg).

The incidence of patients with KD who do not respond to initial treatment with IVIG is approximately 10% to 20% [4]. Our research shows that the incidence of IVIG resistance is about 8.38%. A large amount of research suggests that the incidence of CALs in IVIG-resistant KD is as high as 22%-45.8% [12, 16]; our study shows that 33 of 80 IVIG-resistant patients had CALs, an incidence of 41.25%. Because the incidence of CALs is so high, it is important to find a more beneficial treatment for these children. The current retreatment options mainly use additional IVIG, corticosteroids, and infliximab, among others [11, 17–19]. Several retrospective study results suggest that MPT for IVIG-resistant KD may reduce the risk for CALs [20], but there are no robust data from clinical trials to guide the clinicians in the choice of therapeutic agents for the children with IVIG resistance [13].

Corticosteroids were used as the initial therapy for KD long before the first report by Furusho et al. in 1984 [21]. Some studies reported that steroid treatment for KD is unsafe and contraindicated due to the high incidence of CALs [22]. Recently, because of its cost-effectiveness, MPT for IVIG-resistant KD has increasingly attracted clinicians' attention [23]. Shinohara et al. [24] found that prednisolone led to a lower prevalence of coronary artery aneurysms and could significantly shorten fever duration. A recent meta-analysis by Chen et al. [25] found that a combination of corticosteroids with standard-dose IVIG as an initial treatment in high-risk patients could reduce the rate of CALs. There is still no convincing research on the clinical efficacy of MPT on KD. In particular, the long-term effect of MPT on coronary arteries has not been clarified [26].

In our trial, comparing an IVIG retreatment group and an MPT group, we found that patients in the MPT group had a shorter duration of fever after retreatment and lower medical costs; a more rapid decline in CRP, N%, and PLT levels; and a more rapid rise in sodium. Moreover, they were not significantly different in terms of preventing the development of CALs in the short-term follow-up (≤6 mo). However, our research found that the MPT group probably had a higher incidence of CALs than did the IVIG treatment group in the long-term follow-up (>6 mo).

The optimal steroid regimen is, therefore, not known, and both pulsed and longer-term steroid therapies remain options. Furukawa et al. [7] analyzed the effects of IVMP pulse therapy (methylprednisolone (30 mg/kg/day) was administered over 2 h for 3 consecutive days, and prednisolone (1 mg/kg/day) was administered and tapered over 7 days as aftercare) compared with additional IVIG (2 g/kg) in IVIG-resistant patients. They found that IVMP is an effective additional treatment for IVIG-resistant KD. However, there was a tendency for fever to recur later in IVMP-resistant patients, which could potentially delay the therapeutic decision-making process. Miura et al. [27] conducted a control study in KD patients with persistent or recurrent fever (>37.5°C) 48 hours after a single infusion of initial intravenous immunoglobulin (IVIG) 2 g/kg. At enrolment (day 1), the subjects were randomly to receive IVMP (30 mg/kg/day of methylprednisolone for three days), or additional IVIG (2 g/kg). They concluded that KD patients refractory to initial IVIG should be treated with additional IVIG, because IVMP induced faster but temporary resolution of fever and more adverse effects, and further investigations with steroid therapy are necessary to determine the indication and the appropriate dose in KD. Lang et al. [18] found that corticosteroids are effective in the treatment of fever in most patients with IVIG-refractory KD, but a multicenter prospective study is needed to determine the effect of corticosteroids on CAA outcome in patients with refractory KD. In our study, we were concerned about the side effects of high-dose intravenous hormones, so we gave intravenous methylprednisolone (15 mg/kg/d) for 3 d consecutively, without a subsequent course and taper of oral prednisone. This study suggests that patients in the MPT group had a shorter duration of fever after retreatment and lower medical costs; a more rapid decline in CRP, N%, and PLT levels; and a more rapid rise in sodium, but had a higher incidence CALs than patients in the second IVIG treatment group in long-term follow-up (>6 mo). However, we discovered that the readmission rate in patients treated with MPT was significantly higher than that in patients treated with additional IVIG treatment. We think the high readmission rate may be related to the sudden withdrawal of intravenous methylprednisolone and lack of a subsequent course and taper of oral prednisone.

The guideline [13] suggests the administration of high-dose pulse steroids for retreatment of patients with KD who have had recurrent or recrudescent fever after additional IVIG, but one study suggests that high-dose MPT may cause bradycardia and elevated blood pressure and blood sugar [27]. Consequently, clinicians are more cautious about the application of MPT to IVIG-resistant KD, and more ordinary doses are used. The dose of methylprednisolone used in our study was 15 mg/kg/d for 3 consecutive days, and although five patients developed bradycardia in the MPT group, their conditions improved spontaneously.

Immunoglobulin is expensive and, as a blood product, poses risks related to blood transfusion. The anti-inflammatory effect of corticosteroids is certain, and their medical cost is relatively low. A comprehensive analysis of cost-effectiveness shows that there is a bright future for IVIG-resistant KD [28]. Our study shows that the MPT group had a shorter duration of fever after retreatment and lower medical costs; a more rapid decline in CRP, N%, and PLT levels; and a more rapid rise in sodium in the treatment of IVIG-resistant KD. However, the study also shows that the readmission rate in patients treated with MPT was significantly higher than that in patients treated with additional IVIG, and the MPT group probably had a higher incidence of CALs in long-term follow-up. Therefore, caution is required in the use of MPT to treat IVIG-resistant KD.

Although this study is the largest to be performed with Chinese children and the first prospective RCT for patients with IVIG-resistant KD in China, certain limitations should be acknowledged. First, the sample size in the current study is still small, and multicenter prospective RCTs with larger sample sizes are needed. Second, we only compared the efficacy and safety of retreatment of IVIG therapy with MPT (15 mg/kg/d for 3 consecutive days, without a subsequent course and taper of oral prednisone) in patients with IVIG-resistant KD. Many other therapies were not compared, such as MPT (20-30 mg/kg/d for 3 d consecutively, with a subsequent course and taper of oral prednisone). These studies will be conducted in the future.

## Figures and Tables

**Table 1:** Age, sex, inpatient day, duration of fever, duration of fever after retreatment, and medical cost in both groups.

	Group A (*n* = 40)	Group B (*n* = 40)	95% CI	*P* value
Age (mo)	21.0 ± 14.4	25.6 ± 21.1	0.48-1.67	0.178
Sex (male : female)	28 : 11	28 : 13	0.63-1.37	0.998
Weigh (kg)	10.7 ± 3.2	11.7 ± 4.7	0.13-0.57	0.131
Hospital day (d)	6.7 ± 2.6	6.2 ± 2.3	0.27-0.83	0.233
Duration of fever (d)	11.2 ± 2.3	10.3 ± 2.0	0.21-2.60	0.196
Duration of fever after retreatment (h)	18 ± 4.4	11 ± 6.3	0.11-0.70	0.026
Medical cost (yuan)	¥11261 ± 3564	¥8218 ± 2145	0.19-0.81	0.018
Readmission rate	10.0% (4/40)	27.5% (11/40)	0.23-0.76	0.026

Data are expressed as the mean ± SD. Group A: IVIG retreatment group; Group B: MPT group.

**Table 2:** Laboratory data on additional treatment.

		Group A (*n* = 40)	Group B (*n* = 40)	95% CI	*P* value
WBC (10^9^/L)	Before therapy	16.1 ± 6.9	18.7 ± 6.9	0.50-3.98	0.292
	After therapy	10.7 ± 4.2	11.6 ± 5.3	0.71-1.57	0.087
	△WBC	5.2 ± 7.2	7.0 ± 5.9	0.50-3.94	0.247

N (%)	Before therapy	54.4 ± 11.9	56.5 ± 14.7	0.06-3.58	0.053
	After therapy	40.4 ± 15.0	44.2 ± 14.1	0.06-3.99	0.127
	△neutrophils	12.5 ± 15.0	14.6 ± 14.0	0.27-0.84	0.048

CRP (mg/L)	Before therapy	100.4 ± 56.3	123.1 ± 69.8	0.19-7.25	0.652
	After therapy	16.0 ± 25.2	21.9 ± 31.5	0.28-6.99	0.725
	△CRP	80.5 ± 46.8	101.5 ± 64.7	0.10-0.52	0.039

HGB (g/L)	Before therapy	92.8 ± 11.3	94.9 ± 13.3	0.27-1.57	0.125
	After therapy	102.2 ± 13.5	101.0 ± 13.5	0.28-1.57	0.265
	△HGB	9.1 ± 15.0	6.1 ± 12.4	0.25-1.32	0.114

PLT (10^9^/L)	Before therapy	447 ± 210.5	408 ± 176.8	0.6-3.72	0.458
	After therapy	599.9 ± 204.7	647.2 ± 229.7	0.52-1.20	0.850
	△PLT	149.0 ± 254.8	238.6 ± 291.5	0.13-0.57	0.027

Albumin (g/L)	Before therapy	92.8 ± 11.3	94.9 ± 13.3	0.51-1.19	0.232
	After therapy	102.2 ± 13.5	101.0 ± 13.5	0.52-1.20	0.335
	△albumin	3.8 ± 5.4	5.4 ± 6.7	0.19-0.80	0.110

Sodium (mmol/L)	Before therapy	130.8 ± 19.5	134.1 ± 2.9	0.30-7.25	0.630
	After therapy	137.3 ± 1.9	138.7 ± 2.1	0.28-6.99	0.095
	△sodium	3.3 ± 2.6	4.8 ± 2.8	0.27-0.83	0.043

Data are expressed as the mean ± SD. WBC = white blood cells; HGB = hemoglobin; CRP = C-reactive protein; PLT = platelets; Group A: IVIG retreatment group; Group B: MPT group. Before therapy: values 1 d before the 2nd treatment. After therapy: values 7 d after the 2nd treatment. **△**: difference in laboratory data were calculated using the equation values 7 d after the 2nd treatment − values 1 d before the 2nd treatment.

**Table 3:** CALs in the two groups.

Follow-up time	Group A (*n* = 40)				Group B (*n* = 40)				*X*_2_	*P*
	Dilation	SA	MA	GA	Dilation	SA	MA	GA	CALs	CALs
7 days (*n* = 80)	5/40	8/40	2/40	0/40	6/40	9/40	3/40	0/40	0.464	0.650
1 month (*n* = 79)	2/39	5/39	1/39	0/39	2/40	4/40	3/40	1/40	1.939	0.202
3 months (*n* = 78)	3/39	0/39	1/39	0/39	0/39	0/39	2/39	1/39	0.157	1.000
6 months (*n* = 71)	2/35	0/35	0/35	0/35	0/36	2/36	1/36	1/36	0.668	0.674
12months (*n* = 53)	0/28	0/28	0/28	0/28	0/25	2/25	1/25	1/25	4.846	0.043
24months (*n* = 33)	0/18	0/18	0/18	0/18	0/14	2/15	2/15	0/15	5.462	0.033

SA: small aneurysm; MA: medium aneurysm; GA: giant aneurysm; CALs = dilation + SA + MA + GA.

## Data Availability

The data used to support the findings of this study are included within the article. The authors confirm that the data supporting the findings of this study are available within the article.

## References

[1] Kawasaki T. (1967). Acute febrile mucocutaneous syndrome with lymphoid involvement with specific desquamation of the fingers and toes in children. *Arerugī*.

[2] Daniels L. B., Tjajadi M. S., Walford H. H. (2012). Prevalence of Kawasaki disease in young adults with suspected myocardial ischemia. *Circulation*.

[3] Terai M., Shulman S. T. (1997). Prevalence of coronary artery abnormalities in Kawasaki disease is highly dependent on gamma globulin dose but independent of salicylate dose. *The Journal of Pediatrics*.

[4] Newburger J. W., Sleeper L. A., McCrindle B. W. (2007). Randomized trial of pulsed corticosteroid therapy for primary treatment of Kawasaki disease. *The New England Journal of Medicine*.

[5] Burns J. C., Capparelli E. V., Brown J. A., Newburger J. W., Glode M. P. (1998). Intravenous gamma-globulin treatment and retreatment in Kawasaki disease. *The Pediatric Infectious Disease Journal*.

[6] Uehara R., Belay E. D., Maddox R. A. (2008). Analysis of potential risk factors associated with nonresponse to initial intravenous immunoglobulin treatment among Kawasaki disease patients in Japan. *The Pediatric Infectious Disease Journal*.

[7] Furukawa T., Kishiro M., Akimoto K., Nagata S., Shimizu T., Yamashiro Y. (2008). Effects of steroid pulse therapy on immunoglobulin-resistant Kawasaki disease. *Archives of Disease in Childhood*.

[8] Sundel R. P., Burns J. C., Baker A., Beiser A. S., Newburger J. W. (1993). Gamma globulin re-treatment in Kawasaki disease. *The Journal of Pediatrics*.

[9] Newburger J. W., Takahashi M., Gerber M. A. (2004). Diagnosis, treatment, and long-term management of Kawasaki disease. *Circulation*.

[10] Inoue Y., Okada Y., Shinohara M. (2006). A multicenter prospective randomized trial of corticosteroids in primary therapy for Kawasaki disease: clinical course and coronary artery outcome. *The Journal of Pediatrics*.

[11] Wallace C. A., French J. W., Kahn S. J., Sherry D. D. (2000). Initial intravenous gammaglobulin treatment failure in Kawasaki disease. *Pediatrics*.

[12] Wright D. A., Newburger J. W., Baker A., Sundel R. P. (1996). Treatment of immune globulin-resistant Kawasaki disease with pulsed doses of corticosteroids. *The Journal of Pediatrics*.

[13] McCrindle B. W., Rowley A. H., Newburger J. W. (2017). Diagnosis, treatment, and long-term management of Kawasaki disease: a scientific statement for health professionals from the American Heart Association. *Circulation*.

[14] Carlin J. B., Doyle L. W. (2002). Sample size. *Journal of Paediatrics and Child Health*.

[15] Newburger J. W., Takahashi M., Gerber M. A. (2004). Diagnosis, treatment, and long-term management of Kawasaki disease: a statement for health professionals from the Committee on Rheumatic Fever, Endocarditis, and Kawasaki Disease, Council on Cardiovascular Disease in the Young, American Heart Association. *Pediatrics*.

[16] Kobayashi T., Inoue Y., Takeuchi K. (2006). Prediction of intravenous immunoglobulin unresponsiveness in patients with Kawasaki disease. *Circulation*.

[17] Egami K., Muta H., Ishii M. (2006). Prediction of resistance to intravenous immunoglobulin treatment in patients with Kawasaki disease. *The Journal of Pediatrics*.

[18] Lang B. A., Yeung R. S., Oen K. G. (2006). Corticosteroid treatment of refractory Kawasaki disease. *The Journal of Rheumatology*.

[19] Sonoda K., Mori M., Hokosaki T., Yokota S. (2014). Infliximab plus plasma exchange rescue therapy in Kawasaki disease. *The Journal of Pediatrics*.

[20] Cohen S., Tacke C. E., Straver B., Meijer N., Kuipers I. M., Kuijpers T. W. (2012). A child with severe relapsing Kawasaki disease rescued by IL-1 receptor blockade and extracorporeal membrane oxygenation. *Annals of the Rheumatic Diseases*.

[21] Furusho K., Nakano H., Shinomiya K. (1984). High dose intravenous gammaglobulin for Kawasaki disease. *The Lancet*.

[22] Hirohisa K., Sigeyuki K., Takashi Y. (1979). Kawasaki disease: effect of treatment on coronary artery involvement. *Pediatrics*.

[23] Teraguchi M., Ogino K. Y., Yoshimura K. (2013). Steroid pulse therapy for children with intravenous immunoglobulin therapy-resistant Kawasaki disease: a prospective study. *Pediatric Cardiology*.

[24] Shinohara M., Sone K., Tomomasa T., Morikawa A. (1999). Corticosteroids in the treatment of the acute phase of Kawasaki disease. *The Journal of Pediatrics*.

[25] Chen S., Dong Y., Yin Y., Krucoff M. W. (2013). Intravenous immunoglobulin plus corticosteroid to prevent coronary artery abnormalities in Kawasaki disease: a meta-analysis. *Heart*.

[26] Iemura M., Ishii M., Sugimura T., Akagi T., Kato H. (2000). Long term consequences of regressed coronary aneurysms after Kawasaki disease: vascular wall morphology and function. *Heart*.

[27] Miura M., Ohki H., Yoshiba S. (2005). Adverse effects of methylprednisolone pulse therapy in refractory Kawasaki disease. *Archives of Disease in Childhood*.

[28] Zhang Y. L., Du Z. D., Fu P. P. (2013). Curative effect observation of different doses of intravenous gamma globulin or methylprednisolone in children with unresponsive Kawasaki disease. *Chinese Journal of Evidence-Based Pediatrics*.

